# Plasma rich in growth factors (PRGF) and leukocyte-platelet rich fibrin (L-PRF): comparative release of growth factors and biological effect on osteoblasts

**DOI:** 10.1186/s40729-022-00440-4

**Published:** 2022-10-03

**Authors:** Laura Baca-Gonzalez, Rebeca Serrano Zamora, Lisa Rancan, Francisco González Fernández-Tresguerres, Isabel Fernández-Tresguerres, Rosa M. López-Pintor, Juan López-Quiles, Isabel Leco, Jesús Torres

**Affiliations:** 1 Department of Dental Clinical Specialties. Faculty of Dentistry, Complutense University, Pza./Ramón y Cajal s/n., 28040 Madrid, Spain; 2 Department of Biochemistry and Molecular Biology. Faculty of Medicine, Complutense University, Madrid, Spain

**Keywords:** Platelet, Bone regeneration, Wound healing, Platelet-rich plasma, PRGF, Platelet-rich fibrin, VEGF, IGF-I, PDGF-AB, IL-1 β

## Abstract

**Purpose:**

To compare the release of platelet-derived growth factor (PDGF), vascular endothelial growth factor (VEGF), insulin-like growth factor (IGF-I) and interleukin 1β (IL-1β) of plasma rich in growth factors (PRGF) and leucocyte platelet-rich fibrin (L-PRF) and to evaluate their biological implication in osteoblasts.

**Methods:**

Blood from 3 healthy volunteers was processed into PRGF, immediate L-PRF (L-PRF 0ʹ) and L-PRF 30 min after collection (L-PRF-30ʹ) and a control group. Growth factors release were analyzed at 7 times by ELISA. Cell proliferation, collagen-I synthesis and alkaline phosphatase activity were assessed in primary cultures of human osteoblasts.

**Results:**

A slower controlled release of IGF-I, VEGF and PDGF was observed in the PRGF group at day 14. A higher synthesis of type I collagen was also quantified in PRGF. L-PRF released significantly higher amounts of IL-1β, that was almost absent in the PRGF.

**Conclusions:**

The addition of leukocytes dramatically increases the secretion of proinflammatory cytokines, which are likely to negatively influence the synthesis of type I collagen and alkaline phosphatase (ALP) by osteoblasts.

## Background

Autologous platelet concentrates (APCs) are used as surgical adjuvants to improve wound healing and tissue regeneration. There are many preparation protocols which result in different end products [1], but the underlying biological mechanism is common to all APCs and involves the release of several growth factors (GFs) and cytokines from the cells—platelets and sometimes leucocytes, depending on the APC—included on a fibrin matrix [2, 3].

Most of these GFs have chemotactic and mitogenic properties that promote cellular functions involved in the early stages of tissue healing. Some GFs significant in bone formation include insulin-like growth factor-I (IGF-I), platelet-derived growth factor (PDGF) and vascular endothelial growth factor (VEGF) [3, 4]. IGF-I is released during platelet degranulation and is ubiquitous in blood. It stimulates preosteoblast proliferation and the synthesis of osteocalcin, alkaline phosphatase, and type I collagen. Therefore, it has a local positive effect on osteoblast proliferation and matrix secretion [2, 4, 5]. PDGF-AB can be synthesized by platelets and macrophages and has mitogenic, chemotactic and anti-inflammatory activity and a major role in epithelialization. It can also activate neutrophils and promote TNF-β [2, 4, 6, 7]. VEGF, on its hand, is secreted by platelets, macrophages and neutrophils and promotes the early stages of angiogenesis and endothelial cell migration and proliferation [6, 8, 9].

The fibrin matrix is a scaffold for angiogenesis and cell migration and acts as a carrier for the diffusion of GFs to the local environment [3, 8, 10]. Their release is influenced by their physicochemical properties and by the way proteins bind to the fibrin matrix. Thus, it has been suggested that fibrin release properties are not the same in all APCs [11].

APCs can be classified into two main groups in relation to the characteristics of the fibrin matrix: platelet-rich plasma (PRP) and platelet-rich fibrin (PRF). If, in addition, the leukocyte content is taken into account, the following types of APCs can be distinguished: pure PRP (P-PRP), leukocyte-rich PRP (L-PRP), pure PRF (P-PRF) and leukocyte-rich PRF (L-PRF) [2, 3, 8].

Plasma rich in growth factors (PRGF), which is a type of P-PRP, and L-PRF are common APCs in different medical disciplines due to their simplicity in terms of centrifugation time and ease of preparation. Also, the protocols for L-PRF and PRGF have not undergone major modifications since their introduction. Both platelet concentrates have been tested in different clinical applications, such as treatment of postextraction socket, sinus floor elevation, vertical and horizontal bone augmentation, as well as improvement of osseointegration of dental implants.

The effect of APCs on soft tissues and periodontal ligament cells has been extensively studied, but the literature comparing the effect of GFs release kinetics between L-PRF and PRGF in osteoblast cultures is limited [12–15]. Previous studies focus on the effect of a single protocol on osteoblasts and very little research compares different APC protocols [16–19]. The aims of the present study were to characterize the controlled release of PDGF, VEGF, IGF-I and IL-1β from the fibrin matrix of PRGF and L-PRF as well as to evaluate their biological implication in osteoblasts, in terms of cell proliferation, alkaline phosphatase (ALP) activity, collagen-I biosynthesis and interleukin 6 (IL-6) release.

## Methods

### Volunteers

Three healthy volunteers were included in this study, two women and a man ages 25, 28 and 34. All volunteers signed an informed consent prior to their inclusion. The study was performed in accordance with the Helsinki Declaration of 1975 and the study protocol was reviewed and approved by the Ethical Committee for Clinical Trials of the “Hospital Clínico San Carlos” to carry out the study at “Complutense University” (Madrid, Spain) (Protocol number 20/497-E). Exclusion criteria were systemic disease, pregnancy and/or lactation, drugs known to alter platelet count or function within the past 3 months, and patients with abnormal platelet counts.

From each volunteer, 7 tubes of 9 mL were extracted: 2 blue-capped tubes with 3.8% (w/v) sodium citrate (BTI Biotechnology Institute, Vitoria, Spain); 4 red-capped with yellow ring glass-coated plastic tubes (Intra-Lock, Boca Raton, Florida, USA); 1 white-capped tube without additives (BTI Biotechnology Institute, Vitoria, Spain). Samples of each donor were run by duplicate.

**Fig. 1:**
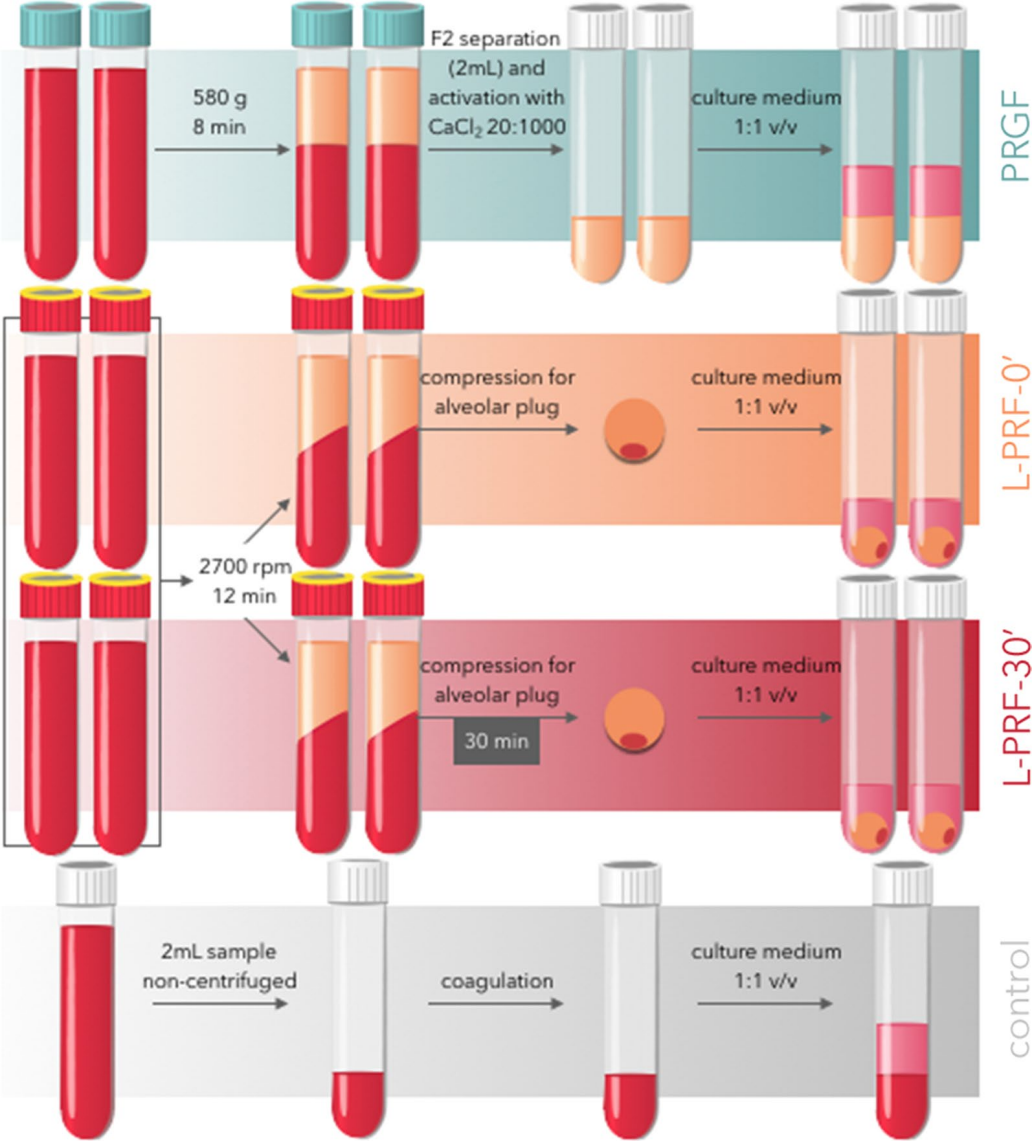
Scheme of the experimental design of the study. The processing of each platelet concentrate is done according to the protocol and manufacturing instructions

Three operators simultaneously carried out each of the protocols following the manufacturers’ instructions. The clot preparation process is illustrated in Fig. 1.

### PRGF preparation

**Fig. 2:**
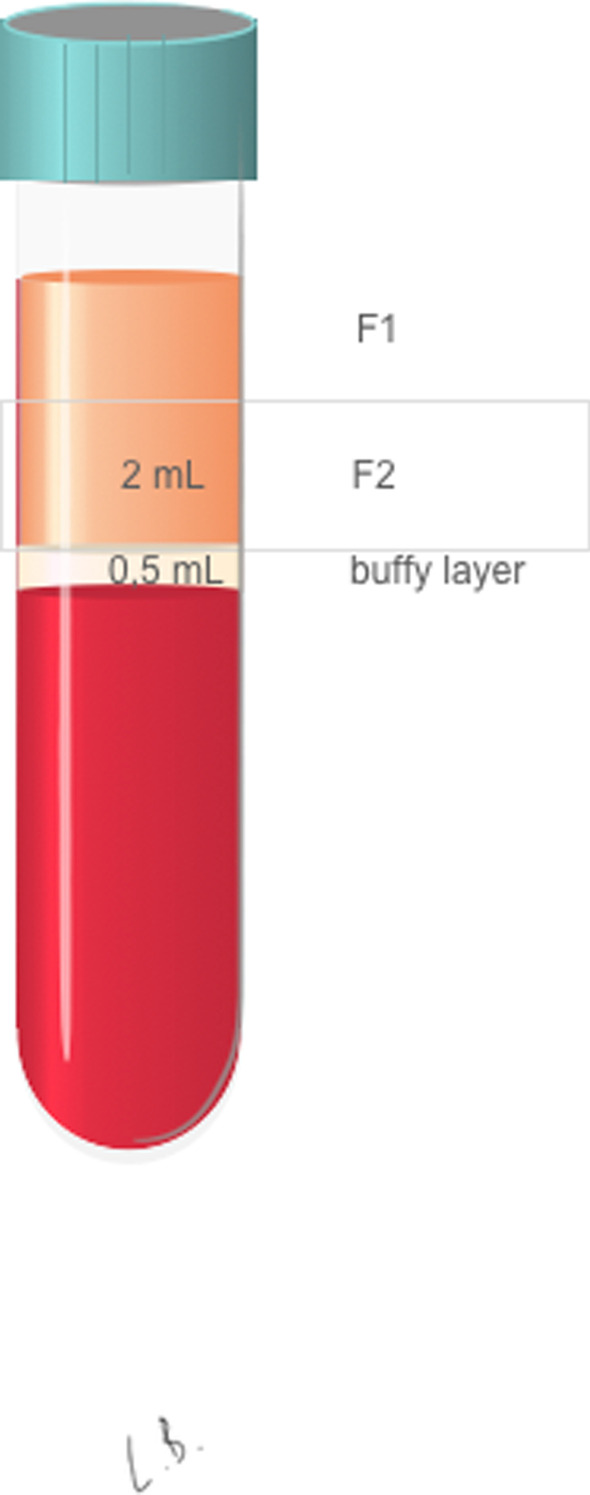
F1: fraction one, consisting in the platelet-poor plasma. F2: fraction two, the 2 mL immediately above the buffy layer, where the platelet concentration is higher

Tubes were centrifuged at 580*g* for 8 min (Centrifuge System V. BTI). The 2 mL immediately above the red fraction is called fraction 2 (F2). F2 has the higher platelet concentration. The leftover above F2 is called fraction 1 (F1) and was discarded. F2 was pipetted, avoiding aspiration of the buffy coat. The F2 was transferred to a labeled tube without additives (BTI) and activated with 20 µL of calcium chloride for every 1 mL of plasma (Fig. 2).

### L-PRF preparation

All the 4 tubes were centrifuged immediately at 2700 rpm for 12 min (IntraSpin, Intra-Lock), count-down starting since the last tube was introduced. Two tubes from each patient were processed immediately after centrifugation (L-PRF 0ʹ) to obtain compressed alveolar plugs in the wells designated for this purpose Xpression^®^ tray; Intra-Lock, Boca Raton, Florida, USA). The fractions were separated by clamping the clot and pressing the limit between the clot and the red series using a non-cutting instrument against the posterior wall of the tube. The excesses of the red phase were removed on a gauze without excessive manipulation to avoid eliminating leukocytes. The remaining two tubes were processed equally 30 min after centrifugation (L-PRF 30ʹ).

### Blood clot preparation

2 mL was separated in a tube without additives and allowed to coagulate. The culture medium was then added.

### Sample processing

Once the clots formed, an equal volume of DMEM/F12 culture medium (Gibco-Invitrogen, Grand Island, New York, USA) with 50 μg/mL gentamicin (Sigma-Aldrich, St. Louis, Missouri, USA) was added. It was then was cultured in an incubator at 37 °C and 5% CO_2_ under saturating humidity conditions. Half the volume of medium was collected and replaced at 1 h, 5 h, 24 h, 48 h, 5 days, 9 days and 14 days after the preparation of the clots. Clot retraction was observed in peripheral blood of donor 3 at 48 h. Changes on the exudate volume due to the said retraction were measured taken into account in the following medium changes and in the calculation of concentrations. The samples from each partial change of medium were centrifuged for 10 min at 460*g* at room temperature to eliminate potential debris and frozen at – 80 °C until its analysis. On day 14, clots were homogenized in 800 μL of culture medium with a Polytron extraction-dispersing machine (Kinematica AG, Lucerne, Lucerne, Switzerland) at 20,000 rpm for 2 min. The suspension was then centrifuged at 21,000*g* for 5 min at 4 °C and the supernatant was stored at − 80 °C until analysis.

### Quantification of growth factors and IL-1beta

Quantification was performed by ELISA for IGF-I (Human IGF-I Quantikine ELISA Kit. DG100. R&D Systems, detection range = 0.1–6 ng/mL; sensitivity = 0.02 ng/mL); PDGF-AB (Human PDGF-AB Quantikine ELISA Kit. DHD00C. R&D Systems, Minneapolis, USA, detection range = 15.6–100 pg/mL; sensitivity = 3.83 pg/mL); VEGF (Human VEGF ELISA kit. KHG0111. Invitrogen, Thermo Fisher Scientific, California, USA, detection range = 23.4–1500 pg/mL; sensitivity < 5 g/mL), and IL-1β (Human IL-1β/IL-1F2. HSLB00D. R&D Systems, range = 0.1–8 pg/mL; sensitivity = 0.063 pg/mL). The kits were used following the manufacturer’s instructions and absorbances were measured (iEMS reader MF, Thermo Labsystems Inc., Beverly, Massachusetts, USA).

### Osteoblast proliferation comparative

Osteoblast culture was carried out according to previous literature [16–18, 20]. Primary alveolar osteoblasts were obtained from the mandible of one healthy 60-year-old female donor during implant surgery, after signing pertinent informed consent. The patient underwent antibiotic prophylaxis 1 h prior to surgery with 2 g of amoxicillin. Bone was harvested using a low-speed drilling protocol (125 rpm) and cultured in phosphate-buffered saline (PBS) containing 50 mg/mL gentamicin and 2.5 mg/mL amphotericin B (Sigma-Aldrich) in a humidified atmosphere at 37 °C with 5% CO_2_.

Once primary osteoblasts left the explants and reached approximately subconfluence, they were detached with animal origin-free trypsin-like enzyme (Invitrogen) and serially subcultured. Cell viability was assessed by trypan blue dye exclusion (Sigma-Aldrich).

Primary osteoblasts were seeded on 96-well optical bottom black microplates at a cell density of 5000 cells/cm^2^ (1650 cell/well). From the first passage onwards, the culture medium was changed to osteoblast basal medium (ObM) (Sciencell Research Laboratories. CA, USA) supplemented with 50 mg/mL gentamicin (Sigma-Aldrich, MO, USA) and 15% fetal bovine serum (FBS) (Biochrom AG, Berlin, Germany).

After 4 days, the culture medium was discarded and replaced with ObM supplemented with 50 mg/mL gentamicin and 15% the conditioned media of the previous experimental phase at the times of 1 h, 24 h, 5 days, 14 days. 15% of clot lysate was added to another group. Cultures were incubated for additional 96 h. The media from the four replicates were then pooled into a single sample and processed as described in the following section. Finally, wells were washed with PBS and frozen at − 80 °C until use. DNA, representing cell proliferation, was quantified with the CyQUANT cell proliferation assay kit (C7026. Molecular Probes. Invitrogen, Grand Island, NY, USA, detection range from 50 to 50,000 cells in 200 µL) following the manufacturer’s instructions. Sample fluorescence was measured with a fluorescence microplate reader (Twinkle LB 970, Berthold Technologies, Bad Wildbad, Germany).

### Osteoblast activity

Cell conditioned media from the proliferation assay were spun at 500*g* for 10 min and preserved at − 80 °C until the analysis. ALP tissue-nonspecific ALP activity, type I collagen and IL-6 synthesized by the osteoblasts were measured by ELISA following manufacturer’s instructions. (Alkaline phosphatase assay kit (fluorimetric). ab83371. Sensitivity ~ 1 µm. Human pro-collagen I alpha 1 SimpleStep ELISA kit. ab210966. Detection range = 39.06–2500 pg/mL; sensitivity = 5.3 pg/mL. Human IL-6 Catchpoint SimpleStep ELISA kit. ab229434. Detection range = 0.97–2000 pg/mL; sensitivity = 0.4 pg/mL. Abcam. Cambridge, UK).

### Statistical analysis

Statistical analysis of data was performed with SPSS. Normality was assessed with Shapiro–Wilk test. Intragroup differences within consecutive times were studied by Greenhouse–Geisser analysis, followed by a within-subjects contrasts test. For intergroup relations, ANOVA for repeated measures was undertaken and a Bonferroni test was carried for post hoc comparisons. The confidence interval assumed was 95%.

## Results

The results have been expressed as percent cumulative release of GFs and IL-1β (Table 1), which means that the result for a given time is the sum of the concentration of the previous time plus the amount released at the new observed time. The total concentration of each GF was calculated by adding the cumulative release at day 14th and the unreleased GF concentration remaining in the clot (Table 2).

**Table 1:** Cumulative released percentage at day 14th

	Control	PRGF	L-PRF-0ʹ	L-PRF-30ʹ
IGF-I	58.77 (± 4.47)^a^	59.70 (± 3.22)^a^	90.34 (± 2.39)^b^	91.66 (± 4.29)^b^
PDGF-AB	73.23 (± 3.68)^a^	57.21 (± 3.48)^b^	84.41 (± 5.96)^a^	83.90 (± 8.32)^a^
VEGF	37.04 (± 10.63)^a^	47.71 (± 29.59)^a^	70.95 (± 14.38)^a^	65.99 (± 15.13)^a^
IL-1 β	34.52 (± 4.12)^a^	52.16 (± 31.37)^a^	96.53 (± 3.50)^b^	96.51 (± 2.82)^b^

Equal letters show that there are no statistically significant differences within groups. Note that comparisons refer exclusively to data in the same row

**Table 2:** GF total concentration taking in account the cumulative release at day 14th and the residuary clot concentration

	Control	PRGF	L-PRF-0ʹ	L-PRF-30ʹ
IGF-I (ng/mL)	127.34 (± 12.20)^ab^	154.47 (± 51.92)^a^	88.40 (± 18.55)^b^	84.82 (± 9.66)^b^
PDGF-AB (ng/mL)	4.72 (± 1.05)^a^	7.25 (± 1.15)^a^	12.39 (± 7.16)^a^	11.03 (± 9.89)^a^
VEGF (pg/mL)	870.85 (± 200.08)^a^	72.33 (± 50.96)^b^	972.26 (± 301.04)^a^	681.30 (± 425.60)^a^
IL-1 β (pg/mL)	772.18 (± 767.23)^a^	1.75 (± 2.25)^a^	2372.62 (± 1225.01)^a^	2495.07 (± 2772.79)^a^

Equal letters show that there are no statistically significant differences within groups. Note that comparisons refer exclusively to data in the same row

### IGF-I

Looking at the cumulative percentages, PRGF released 30% of its IGF-I within 48 h to day 5. On the 14th day, 60% was reached. However, L-PRF-0ʹ showed a release of 60% on the 5th day and L-PRF-30ʹ released the same percentage in the first 48 h. The retention of IGF-I in the PRGF clot was higher than in both groups of L-PRFs.

Release from the control group was lower than from L-PRF-0ʹ (*p* = 0.0022; *p* = 0.003; *p* = 0.001; *p* = 0.002; *p* < 0.001; *p* < 0.001; *p* < 0.001) and L-PRF-30ʹ (*p* = 0.001 at 1 h; *p* < 0.001 for the rest of the measures) at all times.

**Fig. 3:**
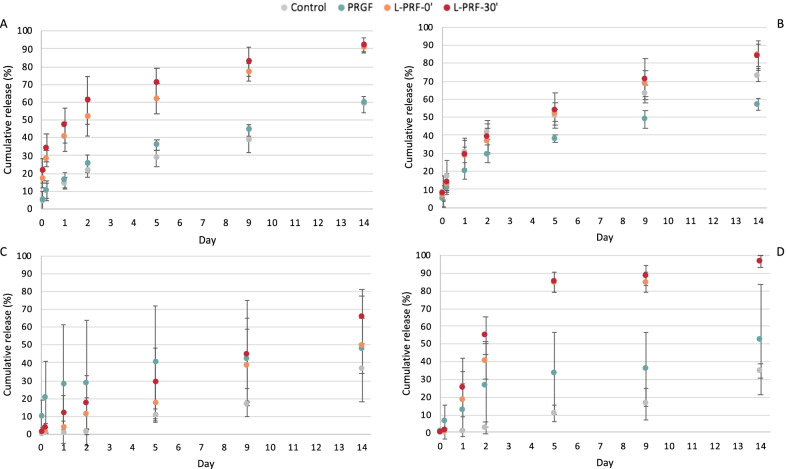
Cumulative release percentage. **A** Insulin-like growth factor (IGF-I). **B** Platelet-derived growth factor (PDGF-AB). **C** Endothelial growth factor (VEGF). **D** Interleukin-1β (IL-1 β)

Moreover, the concentration for PRGF was below L-PRF-0ʹ and L-PRF-30ʹ at all times (L-PRF-0ʹ: *p* = 0.006 at the first hour; *p* = 0.001 at 48 h; *p* < 0.000 was for the rest of the times. L-PRF-30ʹ *p* < 0.001 for all times) (Fig. 3).

Total release percentage is expressed in Table 1.

### PDGF-AB

The total release of PDGF-AB was higher in L-PRF than in PRGF and control, but differences were not statistically significant. Regarding PRGF intragroup cumulative percentage release, we have not observed differences in the first 5 h. However, L-PRFs showed a quick release of PDGF-AB from the 5th day, whereas liberation was slower on PRGF and the control group. While L-PRFs released around 85% at 14 days, PRGF released approximately 60% of total content.

The PDGF-AB release from PRGF was lower than from L-PRF-0ʹ on the 5th, 9th and 14th day (*p* = 0.013; *p* = 0.003; *p* < 0.001) and L-PRF-30’ (*p* = 0.004; *p* = 0.001; *p* < 0.001). PRGF release was also lower than the control at the 14th day (*p* = 0.009) (Fig. 3).

Total released percentage is expressed in Table 1.

### VEGF

The total release of VEGF was higher in L-PRFs than in PRGF. While L-PRF release was around 60%, PRGF liberation was nearly 50%. However, not statistically differences among groups could be found at any time (Fig. 3).

Total release percentage is expressed in Table 1.

### IL-1β

The evaluation of IL-1β concentration in the incubation medium after 14 days showed a significant difference between PRGF and PRFs. The presence of this cytokine was minimally expressed in the samples of PRGF.

From 48 h and onwards, the control group delivered lower amounts of IL-1β compared to L-PRF-0ʹ (*p* = 0.043; *p* < 0.000; *p* < 0.000; *p* = 0.001) and L-PRF-30ʹ (*p* = 0.004; *p* < 0.001; *p* < 0.001; *p* = 0.001). In the same way, there were differences between PRGF compared to L-PRF-0ʹ (*p* < 0.001; *p* < 0.001; *p* = 0.002) and L-PRF-30ʹ (*p* < 0.001; *p* < 0.001; *p* = 0.002) from the 5th day (Fig. 3).

Total release percentage is expressed in Table 1.

### Osteoblasts’ proliferation

Osteoblast proliferative activity was increased in L-PRF-0ʹ (*p* < 0.001) and L-PRF-30ʹ (*p* = 0.001) compared to the control at 24 h. At this time, PRGF was less inductive for the osteoblasts’ proliferation than L-PRF-0ʹ (*p* = 0.008), while L-PRF-30ʹ (*p* = 0.02) had a higher positive effect on cell growth.

**Fig. 4:**
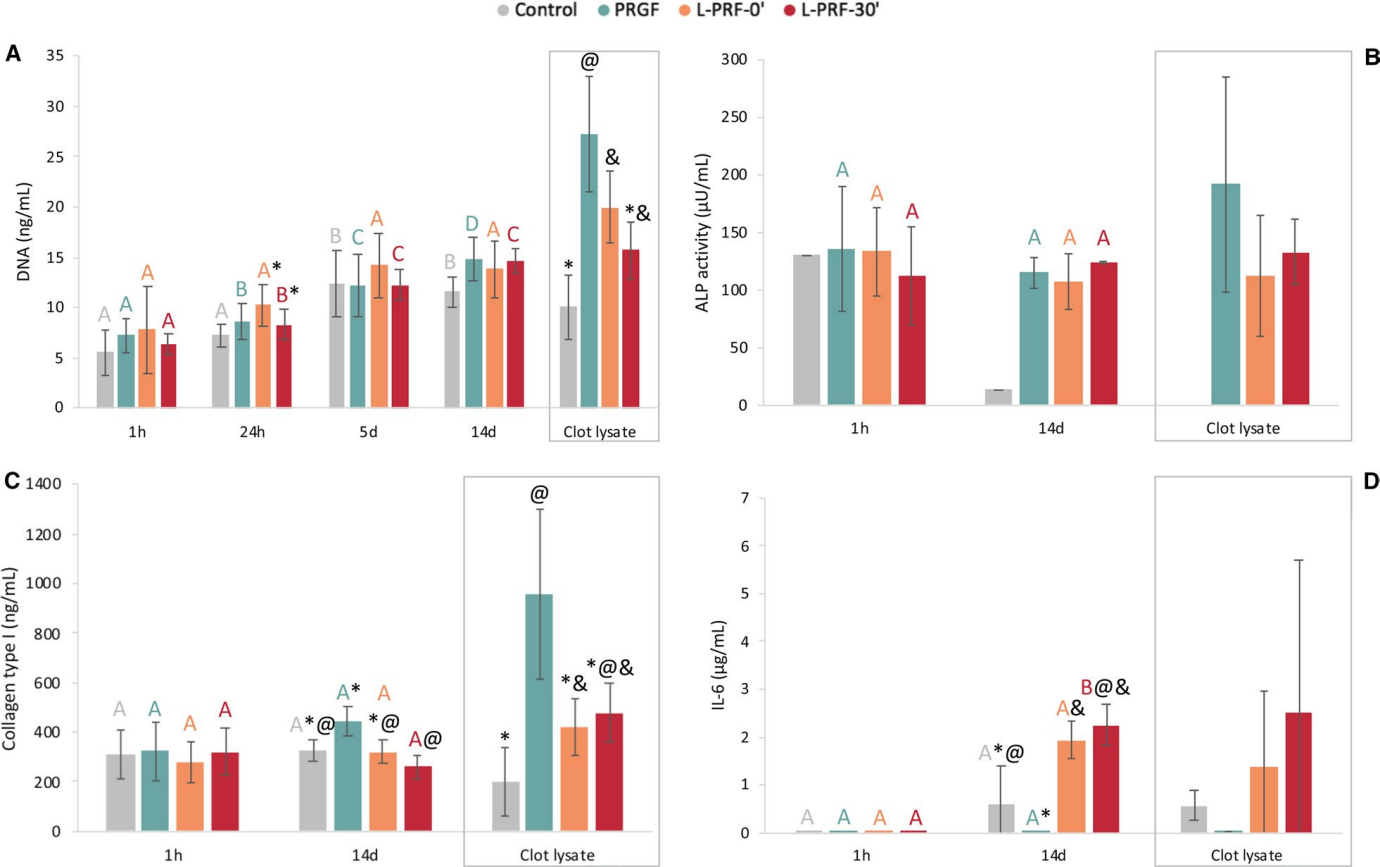
**A** Osteoblast proliferation in terms of DNA release in ng/mL. **B** ALP activity in μU/mL. **C** Collagen type I in ng/mL. **D** IL-6 secreted by osteoblasts in μg/mL. Letters indicate intragroup non-statistically significant differences between two consecutive times. Differences within groups are identified with a symbol. Comparisons of ALP activity could not be stablished on the clot lysate because of a lack of sample on the control group

PRGF clot lysate was the most effective (*p* < 0.001). L-PRF-0ʹ clot lysate improved proliferation compared to the control (*p* = 0.006) (Fig. 4).

### ALP activity

On day 14, control influence on osteoblasts’ ALP activity was remarkably lower than that of PRGF, L-PRF-0ʹ and L-PRF-30ʹ, respectively (*p* = 0.003, *p* = 0.012, *p* < 0.001) (Fig. 4).

### Type I collagen

On day 14th, PRGF release induced a higher type I collagen synthesis than L-PRF-30ʹ (*p* = 0.004). PRGF clot lysate inductive potential was also substantially higher compared to control (*p* = 0.006) and L-PRF-0ʹ (*p* = 0.026) (Fig. 4).

### IL-6

On the 14th day, there was lower IL-6 inductive activity by the control group than L-PRF-0ʹ (*p* = 0.043). Osteoblast IL-6 synthesis was close to null in the presence of PRGF, contrary to the high production triggered by L-PRF-0ʹ (*p* = 0.001) and L-PRF-30ʹ (*p* = 0.003).

No differences were found within unreleased content of IL-6 in the clots between groups (Fig. 4).

## Discussion

Bone regeneration requires a complex coordination between cytokines, proteins and GFs, and the controlled release of these bioactive substances seems to play a key role in this process. This release is modulated by interaction with the extracellular matrix since the retention of GFs by the fibrin network could be related to the binding of GFs to fibrin. In general, release of bioactive substances can occur in three ways: (I) cascade (burst) release of growth factors, (II) rapid release mediated by early degradation of the extracellular matrix, and (III) slow release [21].

Bolus administration of growth factors has limited efficacy and adverse side effects, such as ectopic growth and carcinogenic effects. To overcome these limitations, future research should focus on the development of materials that provide localized delivery and controlled release [21]. Thus, several studies in tissue engineering have used affinity sequestration of growth factor and controlled release to control its delivery over periods ranging from 5 to 25 days. This is of interest in many fields, such as cardiovascular repair, angiogenesis, and bone healing [22–26]. For example, sustained release of VEGF for 3 weeks has been investigated to promote direct capillary formation and blood vessel maturation [25].

Then, the fibrin matrix of APCs would act as a natural regulatory scaffold. Many studies analyze the kinetics of GF’s release from APCs, but there is enormous variability among authors in reporting the results. It has been suggested that these observed differences in the controlled release of GFs in different APCs depend on the architecture of the fibrin matrix and its degree of cross-linking. In this sense, a lower density would imply a faster release [27].

More specifically, previous studies have suggested that the L-PRF fibrin matrix allows a slow and progressive release of GFs over 7 days, whereas the P-PRP fibrin scaffold seems to have a burst release of GFs during the first hours and the rest is released between 3–5 days later [8, 28]. However, this fact was not observed in our samples where, in general, the behavior of PRGF clots was characterized by an initial release of almost 60% during the first 14 days of incubation, with subsequent retention of approximately 40% of GFs within the clot, whereas in PRF clots the release of GFs was almost 90% by day 14.

**Fig. 5:**
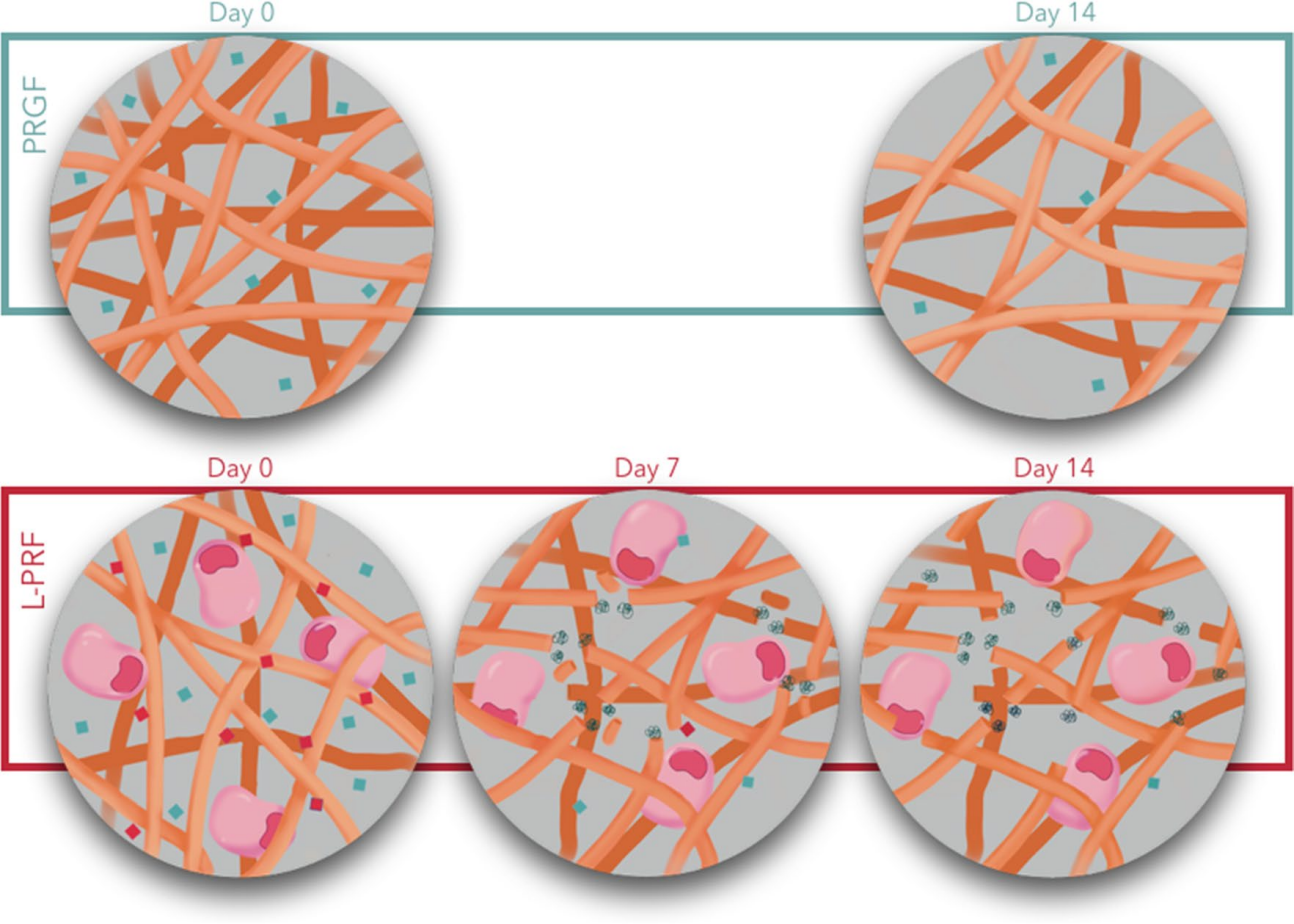
The presence of leukocytes could explain a faster degradation of the fibrin matrix in L-PRF and thus a faster release of growth factors. In addition, the degree of cross-linking of the fibrin mesh could also affect the release kinetics

In view of these results, it would be of great interest to know what happens with this 60% of unreleased GFs in PRGF and whether the controlled release of IGF, VEGF and PDGF beyond 14 days could prolong their beneficial effects on wound healing and improvement of bone regeneration processes. On the other hand, the differences in release dynamics, as observed in this study, could be due to the fact that, perhaps, the degree of cross-linking of the fibrin matrix in PRGF is greater than in L-PRF, contrary to what has been suggested so far in the literature where PRGF membranes even were completely dissolved between 5 and 8 days of incubation [3, 8, 29]. None of the PRGF clots in the present study underwent this process in the observed period. (Fig. 5) Well-designed studies to specifically address long-term behavior are needed.

On the other hand, the presence of leukocytes may be another reason, either jointly or alternatively, to explain the observed differences in release dynamics. There was hardly any IL-1β release in the PRGF group due to the absence of leukocytes in these samples. Leukocytes contain and produce biologically active cytokines that are predominantly catabolic or inflammatory, ROS, and proteases, including collagen-degrading matrix metalloproteinases (MMPs) such as MMP2 and MMP9 [30–33]. Then, matrix degradation would occur more rapidly in L-PRF and, subsequently, a faster release of the proteins bound to it. This is in agreement with what was observed by Anitua et al. in a recent study in which they compared PRGF and L-PRP. They measured d-dimer levels as an indicator of fibrin matrix degradation and found a 179-fold higher concentration in L-PRP clots at 14 days of incubation. They also detected an increased release of collagen from the scaffolds into the culture medium [34].

Furthermore, several studies have also shown a positive correlation between platelet concentration and anabolic gene expression and between leukocytes and catabolic gene expression [32, 33, 35]. Although some studies suggest that defense reactions could be stimulated by the presence of leukocytes and cytokines [8, 36], and hence have a beneficial effect, an excess of leukocytes could overwhelm the ability of GFs to modulate proinflammatory cytokines [32, 33, 35]. There are still no definitive studies comparing platelet-rich plasma with or without leukocytes, but some authors have pointed out the possible negative effects of using platelet-rich plasma with leukocytes in tissue regeneration because a high concentration of proinflammatory cytokines would increase the adverse effects of the inflammatory process, such as pain and swelling [37, 38]. One of the reasons to understand this paradoxical effect could be on the role of macrophages, which are modulators of the immune response, regulating its induction and resolution. These cells show different phenotypes, including proinflammatory macrophages in early stages (M1) and macrophages that favor wound healing (M2) and appear later in the process [33, 36]. M1 cells release proinflammatory cytokines such as interleukin (IL)-1β, IL-6, IL-12, and tumor necrosis factor (TFN) while M2 cells express anti-inflammatory cytokines (IL-4, IL-10), osteogenic signals (TGF-β, bone morphogenetic protein (BMP-2), and angiogenic factors (VEGF and PDGF) which play an essential role in bone regeneration [39–41]. This sequential M1–M2 response results successful for wound healing.

IL-1β has been chosen in this study due to its relevance in bone remodeling, among other functions [42]. In vitro studies have shown that IL-1β directly activates RANK signaling, as well as induces RANKL-mediated osteoclastogenesis and bone loss [43–45]. Also, it induces inflammatory cell infiltration, stimulates MMP production and activates pathways such as NF-kB [46–49]. The L-PRFs groups showed increased IL-1β release with similar kinetics in the cumulative analysis while close to no PRGF release was observed.

However, to determine whether the differences observed in immunohistochemical tests are really relevant to cell function, a second part of the experiment was performed on osteoblast cultures. Osteoblasts are precursor cells of osteocytes. Mature osteoblasts are capable of secreting an osteoid matrix that will later become into a mineralized bone matrix [50]. Osteoblast’s proliferation was determined by DNA quantification while osteoblast maturation and normal function was measured by the synthesis of type I collagen and ALP activity [15, 51]. PRGF on day 14th induced the highest ALP activity, demonstrating the positive effect of PRGF in accelerating the production of extracellular matrix by osteoblasts [52]. These results were also observed for collagen synthesis when PRGF was compared to L-PRF-30ʹ. Also, the clot lysate was more inductive for type I collagen in PRGF than L-PRF-30ʹ. These findings are in agreement with the observed cell proliferation.

At this stage also IL-6 produced by osteoblasts, was analyzed. IL-6 is proinflammatory as well and is closely related to IL-1. In bone tissue, IL-6 stimulates RANKL, which is indispensable for osteoclast differentiation and activation, and its effect would be in conjunction with that of IL-1β, leading to bone resorption and osteoporosis [52, 53]. L-PRF-30ʹ was the strongest promoter of IL-6 at day 14. On the other hand, cells cultured with PRGF showed the lowest IL-6 synthesis.

More studies specifically focused on the inflammatory role of leukocytes are needed to understand the behavioral differences between APCs. Within the limitations of this study, in vitro studies with wider samples and longer follow-up periods are also needed. Moreover, the biological activity of osteoblasts was only evaluated by ELISA, which is essential, but the result from a single method is an important limitation. Further evaluation by PCR with a 14-day approach could be considered for future research.

Also, questions such the role of matrix-degrading enzymes remain unanswered. On the other hand, in vivo clinical trials comparing different APCs effect one bone growth and repair would shed light on the biological importance of our in vitro findings.

The design of this study included 3 young healthy donors, with a very similar profile to each other. In addition, a paired design including a control group was carried out, seeking to increase the homogeneity of the sample.

The applications of this study from the clinical point of view lie in the availability of GFs during the first events of healing. This process in an alveolus occurs in four overlapping stages: (1) hemostasis; (2) inflammatory phase; (3) proliferative phase; (4) remodeling phase.

The first, in which clot formation occurs, takes place immediately after injury. During the second phase, which develops over the next 2 to 3 days, inflammatory cells migrate and the clot begins to colonize with immature fibroblasts and the first vascular buds appear, forming granulation tissue. During the next 2 weeks, fibroblasts mature and this tissue is gradually replaced by collagenous fibers, which corresponds to the proliferative phase. This tissue will subsequently mineralize and remodel during the subsequent months [54, 55].

Our observation period, therefore, would correspond to the first three phases. By controlling the release of proinflammatory cytokines and the release of growth factors, we expect better healing and earlier soft tissue closure that would favor the next steps of healing. This can improve the postoperative period and is especially important in compromised wound healing such as patients with medication or previous pathologies such as diabetes patients at risk of medication-related osteonecrosis of the jaw. Also interesting is the application for the management of surgical complications, such as dehiscence during implant treatment.

We have also verified the beneficial effect of both APCs on osteoblast cultures compared to the control. In this sense, longer release times could favor the initial phases of bone remodeling. This fact also makes the combined use of APCs with particulate grafts particularly interesting. In addition to facilitating their vehiculation, the presence of growth factors from the graft core could perhaps provide some osteoinductive potential to these grafts.

## Conclusions

For all the GFs analyzed, PRGF clots showed an initial release of approximately 50 to 60% during the first 14 days of incubation, whereas in PRF clots IGF-I release was over 90% and 65% for VEGF at day 14. This study suggests important differences in the long-term release of GFs between APCs and this fact could be of importance in extending the benefits of this GFs in wound healing. Furthermore, the addition of leukocytes dramatically increases the secretion of proinflammatory cytokines, which are likely to negatively influence the synthesis of type I collagen and ALP by osteoblasts.

## Data Availability

Not applicable.

## Abbreviations

ALP: Alkaline phosphatase
APCs: Autologous platelet concentrates
BMP: Bone morphogenetic protein
ELISA: Enzyme-linked immunosorbent assay
GFs: Growth factors
IGF-I: Insulin-like growth factor-I
IL-1β: Interleukin-1beta
IL-6: Interleukin-6
L-PRF: Leukocyte- and platelet-rich fibrin
L-PRP: Leukocyte- and platelet-rich plasma
OPG: Osteoprotegerin
PDGF: Platelet-derived growth factor
PRF: Platelet-rich fibrin
PRGF: Plasma rich in growth factors
P-PRF: Pure platelet-rich fibrin
PRP: Platelet-rich plasma
MMPs: Matrix metalloproteinases
NF-kB: Nuclear factor kappa-light-chain-enhancer of activated B cells
P-PRP: Pure platelet-rich plasma
RANK: Receptor activator of nuclear factor k B
RANK-L: Receptor activator of nuclear factor kappa-B
ROS: Reactive oxygen species
TGF-β: Transforming growth factor beta
VEGF: Vascular endothelial growth factor
